# Parallel MR image reconstruction based on triple cycle optimization

**DOI:** 10.1038/s41598-022-11935-w

**Published:** 2022-05-11

**Authors:** Jinhua Sheng, Jie Yin, Luyun Wang, Xiaofan Yang, Pu Huang

**Affiliations:** 1 College of Computer Science, Hangzhou Dianzi University, Hangzhou, 310018 Zhejiang China; 2 Key Laboratory of Intelligent Image Analysis for Sensory and Cognitive Health, Ministry of Industry and Information Technology of China, Hangzhou, 310018 Zhejiang China

**Keywords:** Electrical and electronic engineering, Image processing

## Abstract

The self-calibration parallel imaging (SC-SENSE) method reconstructs the image by estimating the coil sensitivity matrix. In order to obtain the sensitivity matrix, it is necessary to take a small amount of automatic calibration signal lines (ACSL) in the center of *k*-space. This method uses the data of the central region to obtain the sensitivity matrix, and then the reconstructed image is obtained. This paper proposed the triple cycle optimization (TCO) method to continuously optimize reconstructed images. The proposed TCO method takes the sensitivity matrix obtained by ACSL and substituted the reconstructed image as the initial data generation into the loop, and estimates the *k*-space data repeatedly. A new sensitivity matrix is obtained by using *k*-space data and the reconstructed image, and a stable triple cycle is obtained. In the cycle, all data are optimized to a certain extent, including the reconstructed image. Experimental results show that under the same sampling density, images reconstructed by using the triple cycle optimization method have lower noise and artifacts than those of the traditional method. When combined with the variable density sampling method, the effect is remarkable with a much low sampling rate.

## Introduction

Magnetic resonance imaging (MRI), also known as magnetic resonance imaging technology, is another major advancement in medical imaging following Computed Tomography (CT), providing a powerful tool for clinical diagnosis. Compared with CT and ultrasound, MRI can provide more diagnostic information, but the physics of its data acquisition process make it inherently slower than other techniques. During the past 20 years, one of the most important and successful technical developments to decrease MRI scan time was parallel Magnetic Resonance Imaging (pMRI) technology. pMRI is a technology using multi-channel receiving coil information for data acquisition, which greatly reduces the speed of data acquisition. pMRI technology has been a very popular method to shorten the sampling time and accelerate imaging by reducing the sampling data^1–3^. The under-sampling method expands the spacing between the sampling lines, resulting in a reduction in the field of view (FOV), and aliasing artifacts. In order to better reconstruct images based on under-sampled data, researchers have proposed a variety of image reconstruction methods, which are mainly divided into two categories: images reconstructed from undersampled data in either *k*-space or image domain. The representative algorithms based on *k*-space are simultaneous acquisition of spatial harmonics (SMASH)^1^, AUTO-SMASH^4^, VD-AUTO-SMASH^5^, generalized autocalibrating partially parallel acquisition (GRAPPA)^6^, Nonlinear GRAPPA (NL-GRAPPA)^7^, etc., which use specific formulas to estimate missing data in the *k*-space to reconstruct the image. The representative algorithms of image domain-based reconstruction include partially parallel imaging with localized sensitivities (PILS)^8^, sensitivity encoding (SENSE)^9^, self-calibrating parallel imaging with automatic coil sensitivity extraction (SC-SENSE)^10^, joint image reconstruction and sensitivity estimation in SENSE (JSENSE)^11^ and so on. The type of reconstruction methods mainly reconstruct the under-sampled data into aliased images, and then expand aliased images to restore them to clear images. For the latter method, the accuracy of the sensitivity matrix directly affects the quality of the final reconstructed image, so the sensitivity estimation method is as important as the reconstruction algorithm^10^. In recent years, following the success of deep learning in a wide range of applications, neural network-based machine-learning techniques have received interest as a means of MRI^12,13^, which can reduce artifacts and noise in reconstructed images.

Classical parallel imaging in the image space follows the SENSE method. The SC-SENSE method uses variable density to collect the center line, and optimally combines the additional data to improve the image quality^14^. Specifically, in SC-SENSE, sensitivity maps estimation is accomplished by extracting center lines in phase encoding direction in *k*-space transformed by a two-dimensional Fast Fourier Transform (2D FFT) of the intermediate images, according to the automatic calibration signal lines (ACSL) setting^15,16^. To preserve the range the phase-encoding width in *k*-space, other lines are filled with zeros and the inverse Fourier transform of *k*-space data was used to generate a low-resolution, aliasing-free reference image. The standard sensitivity matrix and estimated sensitivity matrix are calculated as follows.

1 $${S}^{standard}=\frac{{M}^{standard}}{{\left(\sum_{i=1}^{N}{\left|{M}^{standard}\right|}^{2}\right)}^\frac{1}{2}}$$

where ${M}^{standard}$ is the image of each coil after full sampling.

2 $${S}^{estimate}=\frac{{M}^{acs}}{{\left(\sum_{i=1}^{N}{\left|{M}^{acs}\right|}^{2}\right)}^\frac{1}{2}}$$

where ${S}^{estimate}$ is the low resolution image of each coil formed by ACSL only sampled.

It can be seen from the above formula that the standard sensitivity is obtained from the image domain data of each coil obtained by full sampling, while the SENSE method is used to reconstruct the image under under-sampling, and the sensitivity matrix is only obtained from the image domain data of the coil formed by Fourier transformation of ACSL in the central region of *k* space. Therefore, there is usually a large truncation error when the sensitivity matrix estimated by ACSL is used to simulate the standard matrix. In particular, when ACSL is small, the information that can be used to obtain sensitivity is scarce, leading to larger truncation errors. In order to solve this problem, many methods have been proposed. The JSENSE method jointly estimates the coil sensitivities and reconstructs the desired image to make a double helix optimization. The proposed method addresses the issue of sensitivity errors by iteratively correcting the sensitivity functions using all acquired variable density (VD) *k*-space data^11^. However, the small amount of data obtained in *k*-space, and the final result may not be well. Thus, we propose a novel method based on triple cycle optimization (TCO) between *k*-space data, sensitivity matrix and reconstructed image. In the cycle, we use the original sensitivity matrix and the reconstructed image to estimate the new *k*-space data, and replace the ACS rows of *k*-space with the information of full sampling in the central region of *k*-space. In this way, the restored *k*-space data contains more information than the original *k*-space with only ACSL data. Therefore, the sensitivity matrix is obtained by using the estimated *k*-space data to reconstruct images accurately. In the cycle, we estimate the complete *k*-space data by optimizing, and increasing the stability between the triple cycles. Since the sensitivity image is smooth, this paper uses the surface fitting function to smooth the sensitivity matrix. In recent years, there have also been methods that combine reconstruction algorithms and acquisition methods to reduce image noise and artifacts, such as PROPOLLER SENSE proposed a new g factor calculation method, which can improve the overall image quality even SNR loss caused by acceleration^17^, MVDS method keeps the number of ACSL in the center of *k*-space unchanged, and VDS of external *k*-space is used for sampling data, which can also improve image reconstruction quality^18^, POCSENSE^19^ and VD-Sense^20^ are also available. In this paper, we applied TCO method to SC-SENSE, and tested and discussed the results of a set of brain data reconstruction. It was discussed whether TCO method can improve the image quality under different collection densities. We combined the multiple variable density sampling (MVDS)^18^ method to compare influences of the TCO method on the experimental results using different fitting functions, and test influences of TCO method on the reconstruction results in the condition of low sampling rate (Supplementary Information).

## Theory

The SC-SENSE algorithm is a reconstruction algorithm based on the image domain. Firstly, the low-resolution and the non-aliasing images are obtained by using ACSL data. These reference images are divided by their sum of squares^10^ to obtain a preliminary estimated sensitivity matrix, which is used as preliminary cycle data. The sensitivity matrix is performed by:

3 $${S}_{0}=Z./{\left(\sum_{i=1}^{N}{\left|Z\right|}^{2}\right)}^\frac{1}{2}$$

where $Z$ is the image domain data from ACSL. ${S}_{0}$ is the sensitivity matrix estimated based only on ACSL.

The sensitivity function matrix is substituted into the SENSE reconstruction algorithm to reconstruct the image. The reconstructed image and the sensitivity function form a new 8-channel image domain data, which is transformed into *k*-space data by Fourier transform.

4 $${D}_{1}(u,v)=\sum_{x=0}^{M-1}\sum_{y=0}^{N-1}({S}_{0}(x,y)*{M}_{0 }(x,y)){e}^{-j2\pi (\frac{ux}{M}+\frac{vy}{N})}$$

where ${M}_{0}$ is the preliminary reconstructed image obtained by bringing ${S}_{0}$ into the SENSE reconstruction algorithm, that is, the reconstructed image obtained by the traditional method.

We use the surface fitting function to constrain the smoothness of the sensitivity matrix. Assuming polynomial surface fitting, the sensitivity function can be expressed as follows:

5 $$S\left(\overrightarrow{r}\right)=\sum_{i=0}^{N}\sum_{j=0}^{N}{a}_{lij}{(x-\overline{x })}^{i}{(y-\overline{y })}^{j}$$

where $(x,y)$ is the position of the pixel, $(\overline{x },\overline{y })$ is the average position, and ${a}_{lij}$ is the coefficient of the polynomial, which is also the coefficient to be sought in the loop optimization.

The experiment tested the sensitivity matrix fitted by the moving least squares method, and compared it with the final result of the polynomial fitting. The moving least squares fitting the sensitivity matrix is given by:

6 $$S\left(\overrightarrow{r}\right)={\sum }_{i=1}^{m}{a}_{i}\left(x,y\right){p}_{i}(x,y)$$

where $a$ is the coefficient to be solved, $p$ is the basis function, which is a complete polynomial of order k, and m is the number of terms of the basis function.

We use the surface fitting function to smoothly constrain the sensitivity matrix, and obtain the coefficients to be solved to obtain the new sensitivity matrix. The formula is as follows:

7 $${D}_{i+1}(u,v)=\sum_{x=0}^{M-1}\sum_{y=0}^{N-1}({S}_{i+1}(x,y)\cdot *{M}_{i}(x,y)){e}^{-j2\pi (\frac{ux}{M}+\frac{vy}{N})}$$

Sensitivity matrix equations in Eqs. (5) and (6) are substituted into Eq. (7). The coefficients are obtained by iteration^19,21^ to form a new sensitivity matrix ${S}_{i+1}$, and ${S}_{i+1}$ is substituted into the reconstruction algorithm to obtain a new reconstructed image ${C}_{i+1}$. By repeating the above steps, the effect of the reconstructed image can be continuously optimized.

**Figure 1:**
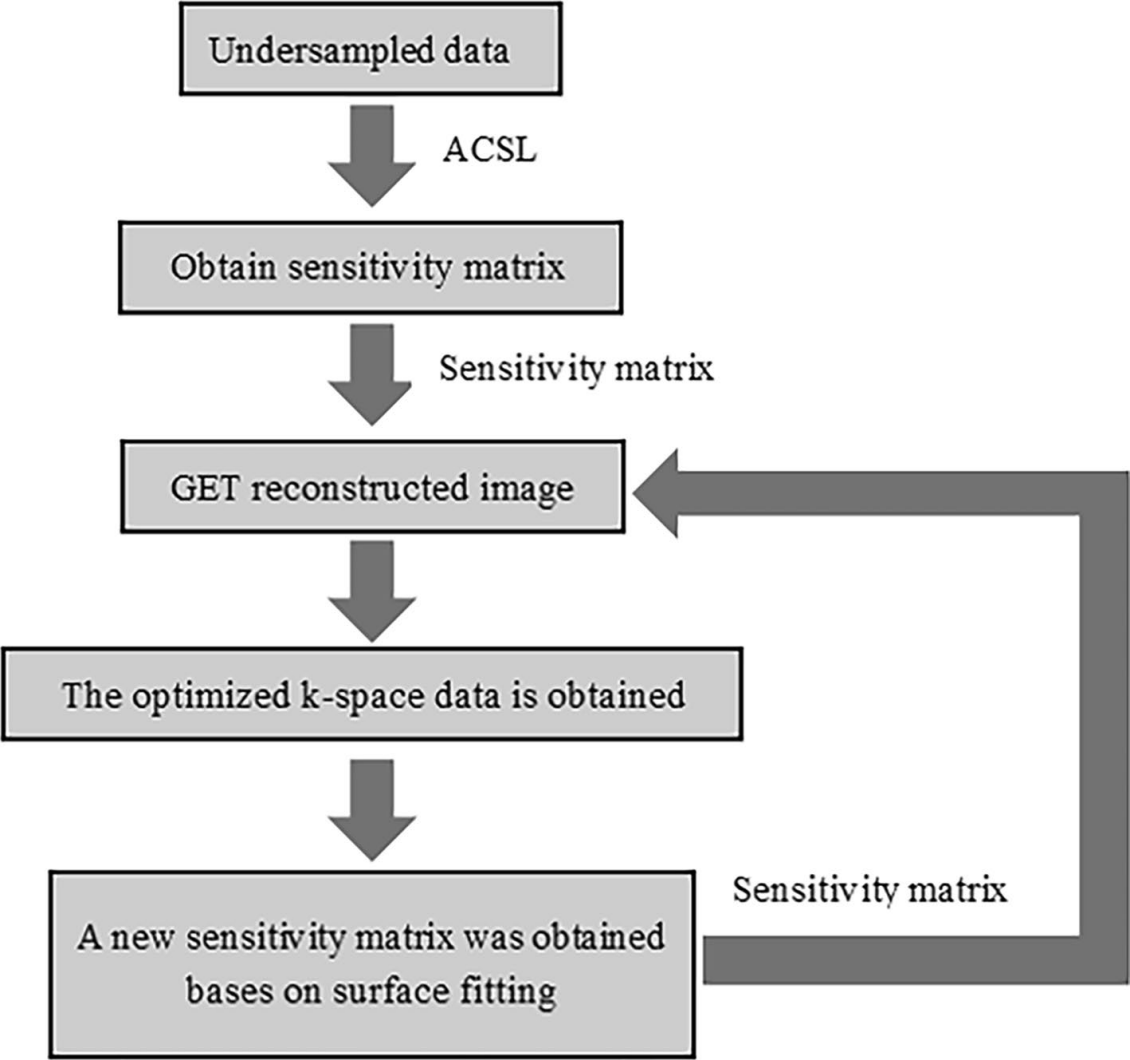
Flow chart of the proposed method.

According to the above steps and formula, the optimized image can be reconstructed, as shown in Fig. 1.

## Materials and methods

The proposed method was implemented in MATLAB. The source data were obtained from https://people.eecs.berkeley.edu/~mlustig/Software.html (University of California, Berkeley, USA) with an 8-channel coil on a 3 T Excite MRI system and gradient echo sequence (TE = 10 ms, TR = 300 ms, RBW = 16 kHz, matrix size = 256 $\times$ 256, FOV = 220 mm $\times$ 220 mm). All data were permitted to publish in an online open access publication. The study was approved by the institutional review board (IRB) at Hangzhou Dianzi University (IRB-2020002) and methods were carried out in accordance with relevant guidelines and regulations.

The complete data set was acquired, and then part of the phase encoding was manually removed to simulate the VD acquisition in pMRI, merge all the data. Reference image is reconstructed by using the sum-of-squares (SoS), which was used as a gold standard for comparison. Using different collection densities, the proposed TCO method was compared with the traditional method. Briefly, the preliminary estimation of sensitivity was made in Eq. (3) using the ACS line data, and then the sensitivity matrix was then smoothed with a curved surface fitting function. Under the ordinary uniform under-sampling method, we adopted the polynomial fitting method (Eq. 5). However, considering that the quality of the reconstructed image under MVDS had been greatly improved, to continue to optimize the image under this sampling method, we compared the effects of polynomial fitting (Eq. 5) and the better fitting function (Eq. 6) on the experimental results. Taking SoS as the standard, the quality of the reconstructed image is quantitatively compared based on the normalized mean square error (RMSE) and signal-to-noise ratio (SNR). The root mean square error was used to describe the error between the reconstructed image and the standard image. The higher value of the RMSE represented increased noise reconstruction, the greater the noise of the reconstructed image is. The calculation method of RMSE was as follows:

8 $$RMSE=\frac{{\sum }_{(x,y)}||{M}^{standard}(x,y)|-|{M}^{reconstruted}(x,y)|{|}^{2}}{{\sum }_{(\mathrm{x},\mathrm{y})}|{M}^{standard}(x,y){|}^{2}}$$

where ${M}^{standard}$ is the standard reference image, and ${M}^{reconstruted}$ is the reconstructed image. According to the Eq. (8), it can be known that the smaller value of $RMSE$ represented the closer the reconstructed image to the standard image, and the smaller error of the reconstructed image.

The image signal-to-noise ratio (SNR) was also an important indicator of image quality. The larger the signal-to-noise ratio represented, the smaller the noise of the reconstructed image. The equation is as follows:

9 $$SNR=10*{log}_{10}\left(\frac{{\sum }_{\left(\mathrm{x},\mathrm{y}\right)}|{M}^{standard}(x,y){|}^{2}}{{\sum }_{(x,y)}||{M}^{standard}(x,y)|-|{M}^{reconstruted}(x,y)|{|}^{2}}\right)$$

## Results

**Figure 2:**
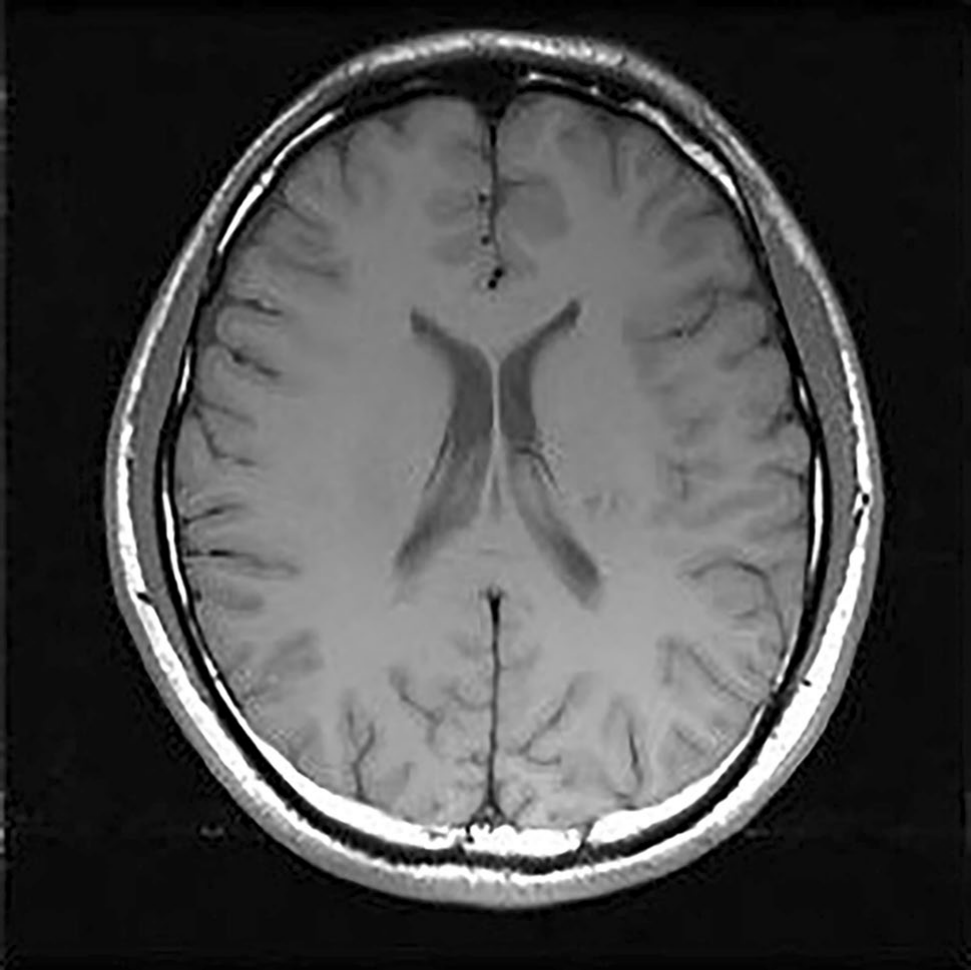
Standard image.

In order to verify the effect of the method proposed in this paper, we used an in-vivo data set. Figure 2 showed the standard reference image obtained from the fully sampled experimental data.

**Figure 3:**
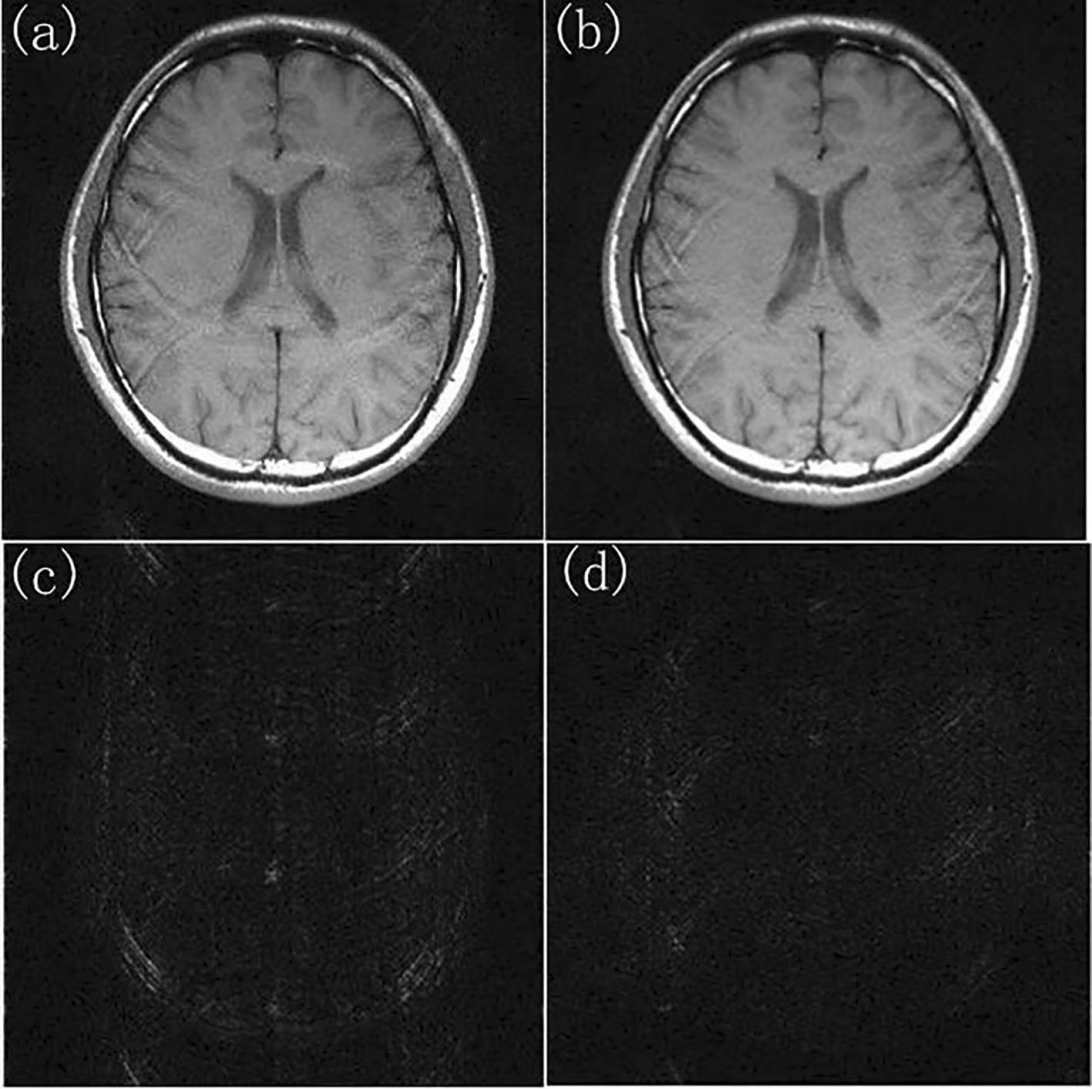
While R = 4, ACSL = 32, the traditional method and TCO method were used to reconstruct images. (**a**) The image reconstructed by using the traditional method, (**c**) the difference map of the image reconstructed by using traditional method, (**b**) the image reconstructed by using TCO method, (**d**) the difference map of the image reconstructed by using TCO method.

**Figure 4:**
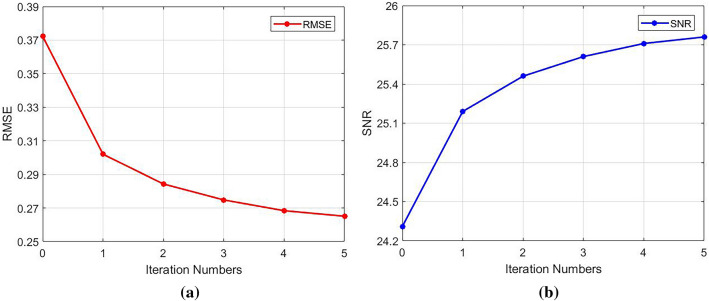
Effect of correction times on image RMSE (**a**) and SNR (**b**) at acquisition density of R = 4 and ACSL = 32.

**Figure 5:**
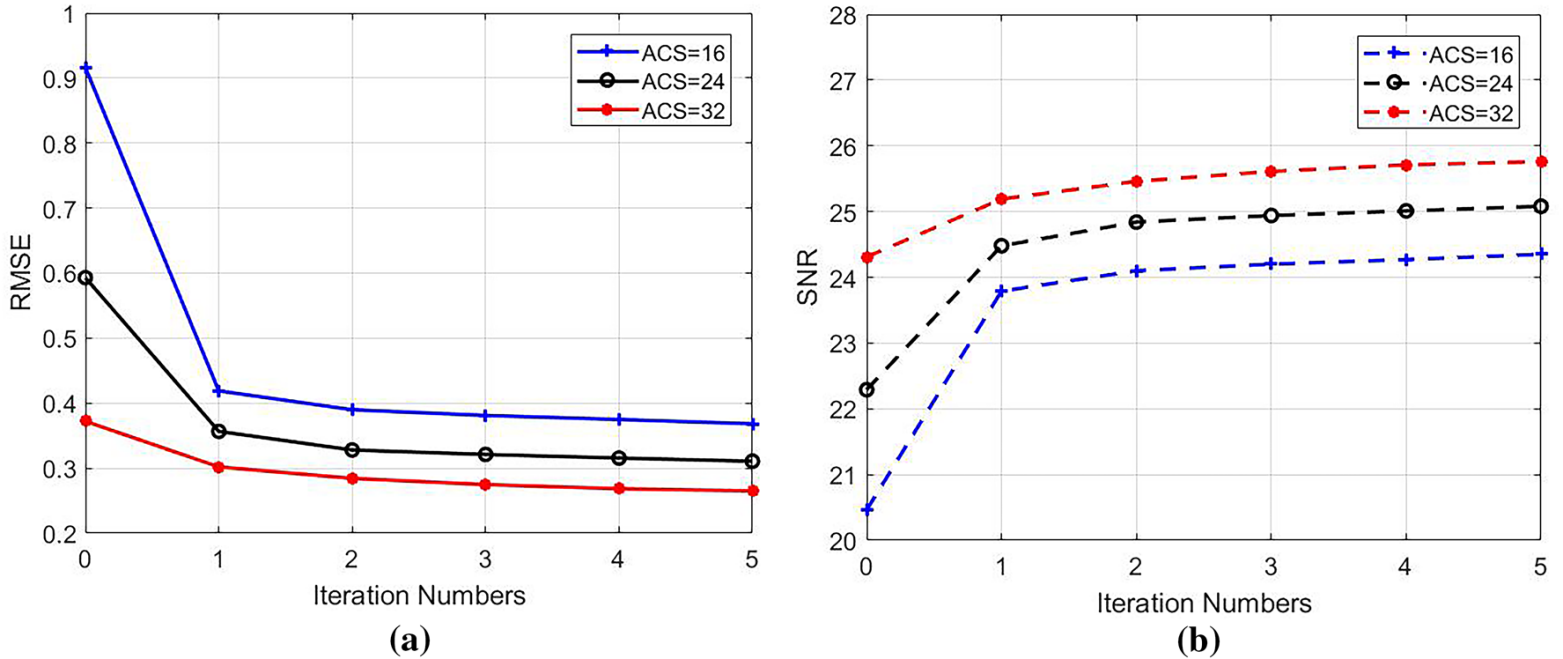
Compare the influence of the number of cycles on the RMSE (**a**) and SNR (**b**) of the reconstructed image under the acquisition density of R = 4 and ACSL = 16, 24, and 32.

The reduced data with different reduction factor (R) and different numbers of ACS lines were used to reconstruct images using different methods. While R = 4 and ACSL = 32, the image reconstructed by using the traditional SENSE algorithm and the difference map between it and the standard image was shown in Fig. 3a,c. Using the proposed TCO method, the sensitivity matrix was fitted with a 6th order polynomial to correct various data. The image reconstructed by using TCO method after the 5th correction and the difference map between it and the standard image was shown in Fig. 3b,d. The corrected root mean square error (RMSE) and signal-to-noise ratio (SNR) ​​were shown in Table 1. We found that the TCO method could effectively improve the quality of the image and reduce the noise of the image. Among them, the 0th time referred to the RMSE and SNR of images reconstructed by using the traditional method without any correction, and the last 5 times were the RMSE and SNR of reconstructed images after continuous correction. It can be seen from Table 1 that with the increase of the number of cycle, the RMSE of reconstructed images showed a downward trend, and the SNR showed an upward trend, indicating that the TCO method had a significant correction effect on reconstructed images. The effects of the number of corrections in the TCO method on the RMSE value and SNR value of the image were shown in Fig. 4a,b. In R = 4, the ACSL ​​were changed to 16 or 24, the multiple experiments were carried out, and final change of the RMSE and SNR were showed in Fig. 4a,b. Under the condition of the same collection density, the results obtained by JSENSE method using the same fitting method have very strong instability. RMSE and SNR of reconstructed images obtained by JSENSE method are shown in Table 2. It can be seen that the quality of image reconstruction deteriorates as the number of cycles increases. This also proves that the JSENSE method has certain instability. While ACSL ​​were changed to 16 or 24, and R = 4, TCO method was used to reconstruct images. The final change of the RMSE and SNR were showed in Fig. 5a,b. It can be seen that no matter which value was taken, with the increase of the number of cycles, the RMSE and SNR ​​had a significant decrease and increase, respectively. It also proved that the proposed TCO method had obvious corrections to the image under these sampling conditions. Especially while ACSL = 16 and R = 4, the TCO method had a significant effect on RMSE.

**Table 1:** Under the condition of R = 4 and ACSL = 32, RMSE and SNR are compared between images reconstructed by using traditional method and TCO method.

	Tradition	TCO method				
	0	1	2	3	4	5
RMSE	0.3722	0.3020	0.2843	0.2748	0.2684	0.2651
SNR	24.31	25.19	25.46	25.61	25.71	25.76

**Table 2:** Under the condition of R = 4 and ACSL = 32, RMSE and SNR are compared between images reconstructed by using traditional method and JSENSE.

	Tradition	JSENSE				
	0	1	2	3	4	5
RMSE	0.3722	0.5126	0.5306	0.5351	0.5348	0.5336
SNR	24.31	22.86	22.71	22.67	22.67	22.68

**Figure 6:**
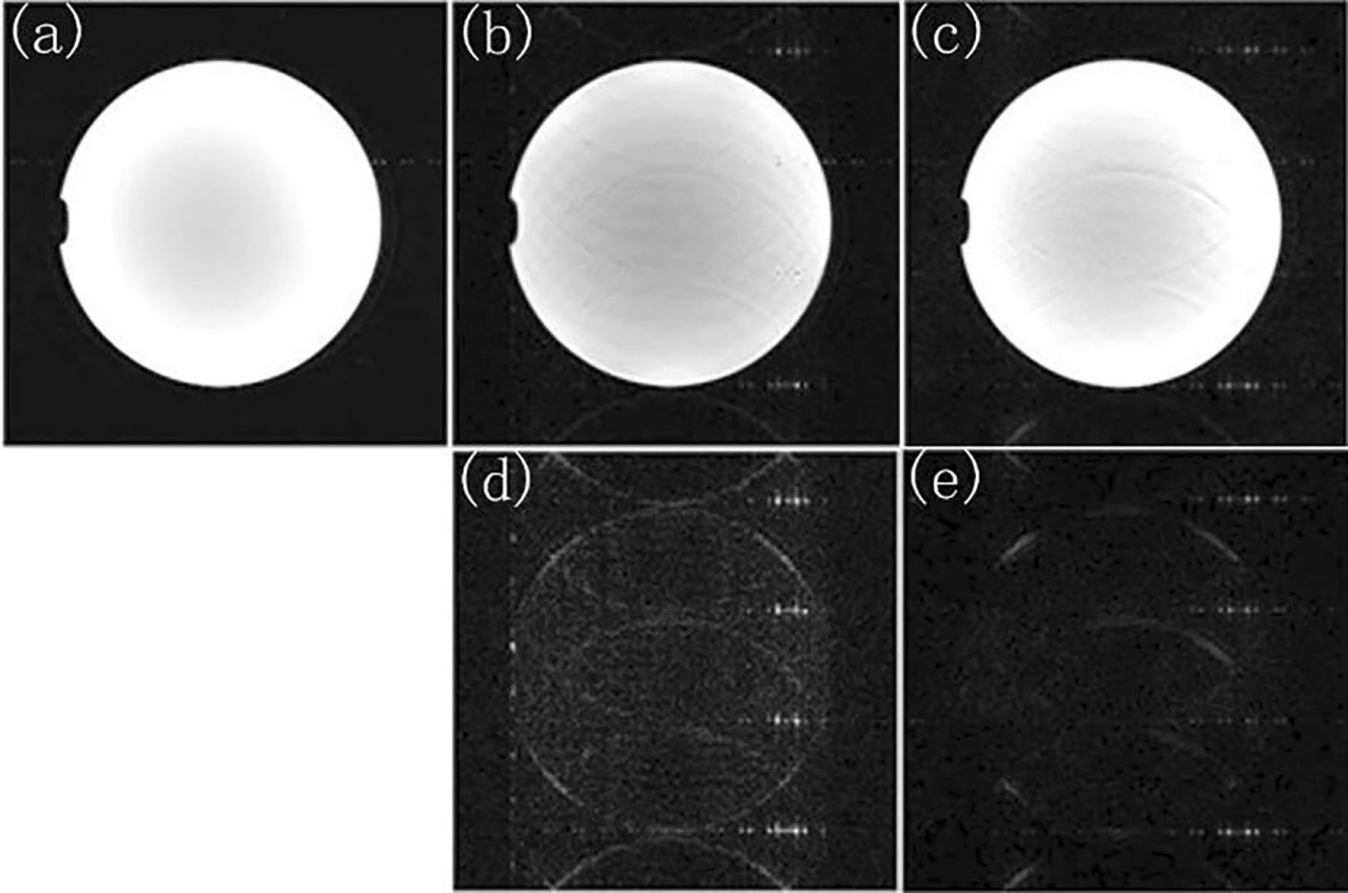
While R = 4, ACSL = 32, the traditional method is used for sampling. (**a**) The standard image, (**b**) the image reconstructed by using traditional method, (**d**) the difference map of the image reconstructed by using traditional method, (**c**) the image reconstructed by using TCO method, (**e**) the difference map of the image reconstructed by using TCO method.

In order to verify the effectiveness of the method, this experiment had been carried out on different data sets. Figure 6a was the standard image. While R = 4 and ACSL = 32, Fig. 6b,d are the image reconstructed by using the traditional method, and the difference map between the reconstructed image and the standard image; Fig. 6c,e are the image reconstructed by using TCO method, and the difference map between the reconstructed image and the standard image. It is obvious that the image reconstructed by using TCO method had less artifact and noise than one of the traditional method. Table 3 showed the root mean square error and signal-to-noise ratio of images reconstructed by using the traditional method and TCO method. It can be seen that the root mean square error of the reconstructed image decreased significantly and the signal-to-noise ratio increased with the number of cycle increases. Therefore, images reconstructed by using TCO method had the higher quality of reconstructed image than those of the traditional method.

**Table 3:** Under the condition of R = 4 and ACSL = 32, the RMSE and SNR of the images reconstructed by using the traditional method and TCO method are compared.

	Tradition	TCO method				
	0	1	2	3	4	5
RMSE	0.1561	0.1364	0.1343	0.1302	0.1277	0.1261
SNR	28.10	28.70	28.77	28.90	28.98	29.03

**Figure 7:**
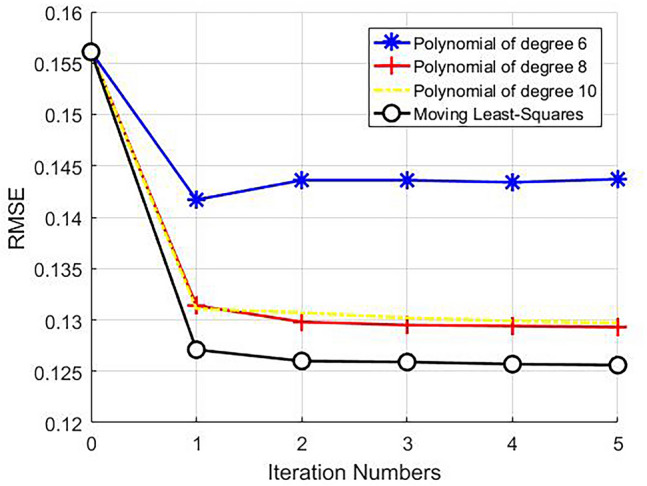
The effect of fitting the sensitivity matrix in different ways.

**Figure 8:**
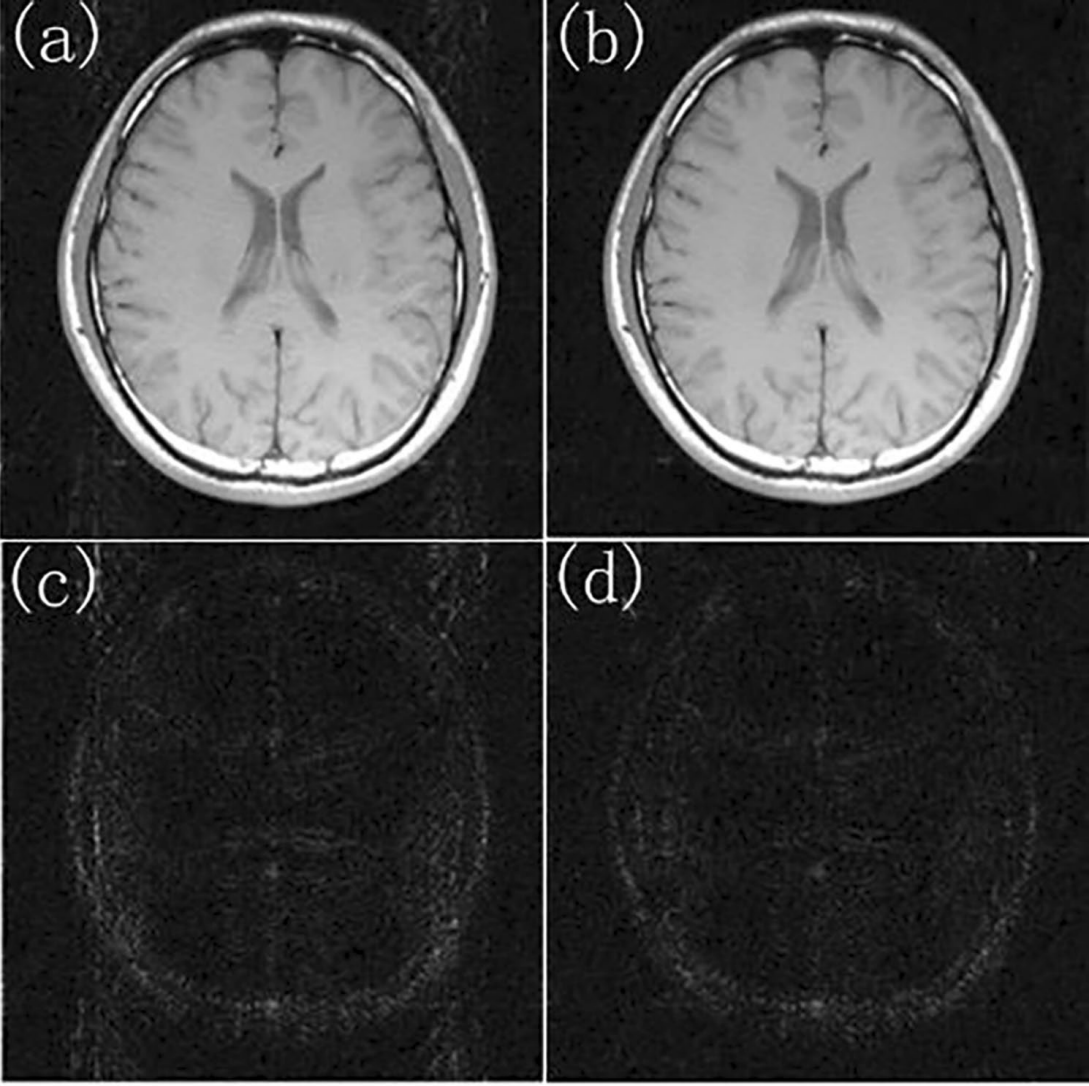
While ACSL = 32 and the total sampling line is 88, the MVDS method is used for sampling. (**a**) Image reconstructed by using the traditional method, (**c**) the difference map of image reconstructed by using the traditional method, (**b**) image reconstructed by using the proposed TCO method, (**d**) the difference map of image reconstructed by using the proposed TCO method.

It can be seen from the above experiments that although the proposed TCO method improved the quality of the reconstructed image, the quality of the reconstructed image was still far from being used. Combining TCO method with MVDS method could improve the image quality on the basis of MVDS. So that it could reconstruct high-quality images under the condition of low ACS. When ACSL = 32 and R = 4, the ACSL data was unchanged and the position of the under-sampling was transformed to improve the image quality. According to the MVDS method, the number of ACSL in the center of the *k*-space was kept unchanged, and the sampled data used the VDS of the external *k*-space. We increased the number of rows with R = 2–38, reduced the number of rows with R = 4–18, and increased the number of rows with R = 6–2. Due to the higher quality of the reconstructed image and smaller errors in nonlinear sampling, the more the surface fitting function fitting the surface represented the better the reconstruction result. At this time, the surface fitting only serves to smooth the sensitivity matrix. Therefore, we compared the effects of moving least squares^19^ and polynomial fitting on the experimental results. Figure 7 described the results of surface fitting with polynomials of degree 6, 8, and 10. It is found that there were fluctuations in the 6th degree polynomial fitting, and the 10th degree polynomial fitting was similar to the 8th degree polynomial fitting. Thus, increasing the polynomial degree had not optimized the experimental results. We used the moving least square method to locally fit the surface, which smoothed the surface and retained the value of the sensitivity matrix to the maximum extent. As shown in Fig. 7, the image reconstructed by the moving least square method was better than that obtained by the polynomial method. Therefore, based on the MVDS method, we used the moving least squares method to fit the sensitivity matrix. The difference between the image directly reconstructed by the MVDS method and the standard image were shown in Fig. 8a,c. The proposed TCO method uses the moving least square method to obtain the image, and the difference map between the reconstructed image and the standard image in Fig. 8b,d. Table 4 showed the transformation of the RMSE and SNR reducing the RMSE value of the RMSE value of the reconstructed image after non-uniform sampling from 0.1561 to 0.1244 by TCO method. It can be seen that the image reconstructed by using TCO method was better than the traditional methods. Therefore, the proposed TCO method based on MVDS optimized the reconstructed image to a large extent, so that it had good reconstruction results when the sampling amount was small. When ACS lines = 24 and under-sampling with, the total sampling lines were 82. Using the MVDS method, the number of sampling lines with R = 2 is set to 40, and the number of sampling lines with R = 4 is set to 16, and the number of sampling rows for R = 6 is set to 2, and the RMSE and SNR values ​​of the obtained image were shown in Table 5. When ACSL = 16 and undersampling with R = 4, the total sampling line obtained is 76. Using the MVDS method, the number of sampling lines with R = 2 is set to 38; the number of sampling lines with R = 4 is set to 16; and the number of sampling rows with R = 6 is set to 2. The RMSE and SNR ​​of the reconstructed images were shown in Table 6. It can be seen from Tables 5 and 6 that TCO method could make the reconstruction quality better, especially when ACSL = 16, the RMSE is reduced from 0.2302 to 0.1414, which is significant.

**Table 4:** The MVDS method is used for sampling when ACSL = 32 and the total sampling line is 88.

	Traditional	TCO method				
	0	1	2	3	4	5
RMSE	0.1561	0.1266	0.1255	0.1249	0.1246	0.1244
SNR	28.05	29.13	29.15	29.20	29.22	29.23

The RMSE and SNR of the images reconstructed by using the traditional method and the proposed TCO method are compared.

**Table 5:** When ACSL = 24 and the total sampling line is 82, the MVDS method is used for sampling.

	Traditional	TCO method				
	0	1	2	3	4	5
RMSE	0.1704	0.1434	0.1390	0.1378	0.1362	0.1350
SNR	27.67	28.77	28.81	28.73	28.78	28.82

The RMSE and SNR of images reconstructed by using the traditional method and the proposed TCO method are compared.

**Table 6:** The MVDS method is used for sampling when ACSL = 32 and the total sampling line is 76.

	Traditional	TCO method				
	0	1	2	3	4	5
RMSE	0.2302	0.1467	0.1432	0.1420	0.1415	0.1414
SNR	26.34	28.50	28.61	28.65	28.67	28.50

The RMSE and SNR of images reconstructed by using the traditional method and TCO method are compared.

**Figure 9:**
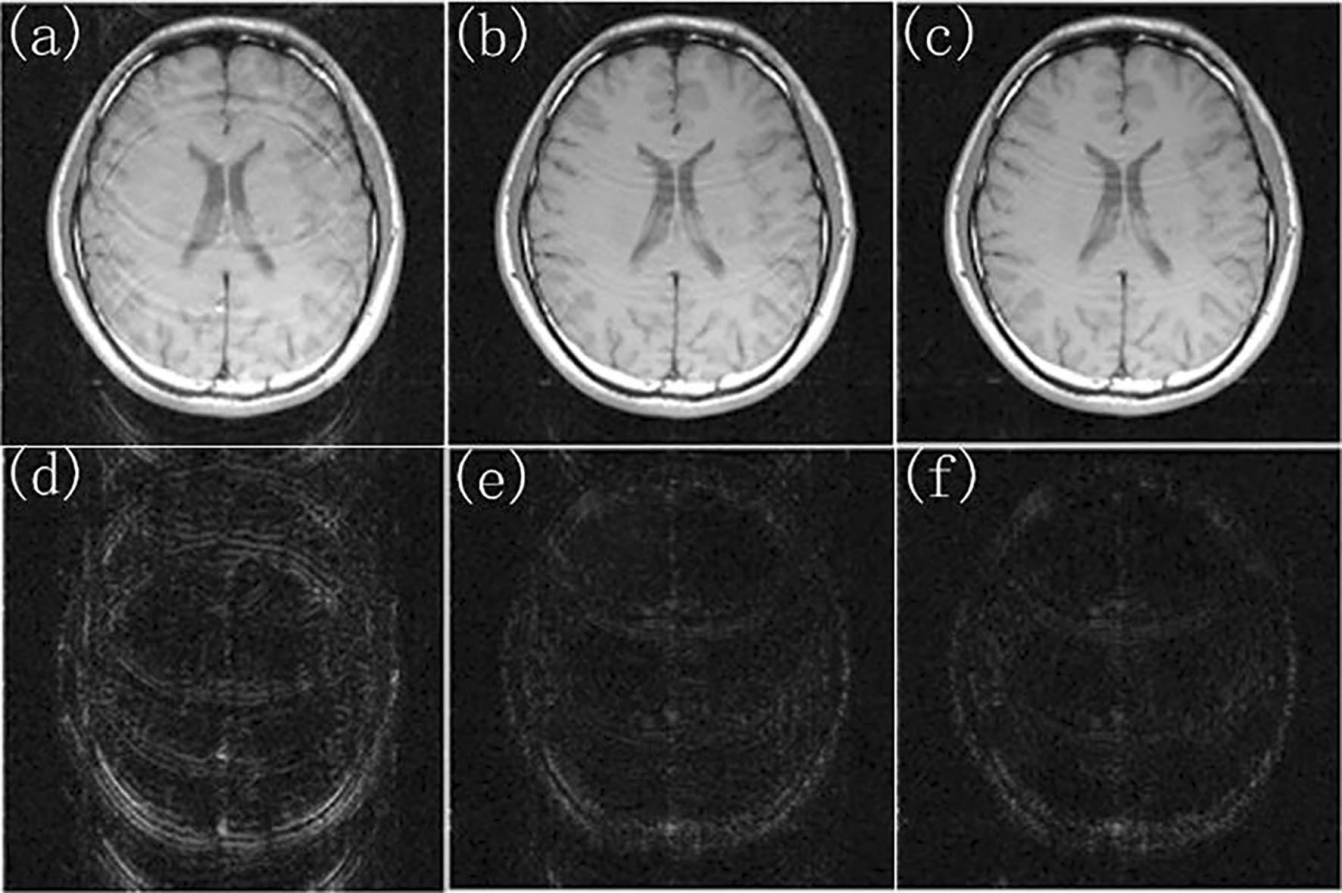
While ACSL = 32 and the total sampling line is 70, the MVDS method is used for sampling. The traditional method is compared with TCO method to reconstruct images. (**a**) The image reconstructed by using the traditional method in uniform undersampling, and (**d**) the difference map of image reconstructed by using the traditional method in uniform under-sampling. (**b**) the image reconstructed by using the traditional method in MVDS sampling, and (**e**) the difference map of image reconstructed by using the traditional method in MVDS sampling. (**c**) The image reconstructed by using TCO method in MVDS sampling, and (**f**) the difference map image reconstructed by using TCO method in MVDS sampling.

Above, we were based on R = 4, in order to reduce the reconstruction time, not only can reduce the ACSL value, but also increase the value of R. Sampling with ACSL = 32, R = 6, the total number of sampling line is 70, Fig. 9a,d showed the difference between the image reconstructed by using traditional method and the standard image with R = 4 uniform under sampling. It can be seen that the reconstructed images have great artifacts and noise. We used MVDS method for sampling, keeping the total sampling line unchanged, increasing R = 2 sampling line to 14, R = 4 sampling line to 22, reducing R = 6 sampling line to 2, Fig. 9b,e showed that the between the image reconstructed by the traditional method and the standard image under this sampling density. Figure 9c,f showed the difference between the image reconstructed by using TCO method and the standard image under the same sampling density. Table 7 showed the RMSE and SNR values ​​obtained under the above conditions. It could be seen that the combination of MVDS and the proposed TCO method improved the reconstructed image even when the total sampling amount was low due to the large value of R. The quality of reconstructed images could be greatly improved under low sampling amount.

**Table 7:** The MVDS method is used for sampling when ACSL = 32 and the total sampling line is 70.

	Traditional	TCO method				
	0	1	2	3	4	5
RMSE	0.2700	0.2129	0.2066	0.2043	0.2034	0.2028
SNR	25.66	26.75	26.88	26.93	26.95	26.96

The RMSE and SNR of images reconstructed by using the traditional method and TCO method are compared.

## Discussion

In this article, we proposed the TCO mode based on the traditional method of obtaining sensitivity matrix. Compared with the traditional methods in which only ACS line data was used to obtain the sensitivity matrix, we proposed to use existing data and methods to achieve a TCO method between *k*-space data, sensitivity matrix and reconstructed image. In this method, after obtaining the sensitivity matrix and reconstructing the image with the traditional method, a new and more accurate *k*-space data was formed with obtained data for subsequent estimation, and then a new sensitivity matrix was estimated by surface fitting function. And the sensitivity matrix was substituted into the SENSE reconstruction algorithm to obtain a new reconstructed image, so as to reciprocate. Experimental results showed that this method had lower noise and artifacts under the same acquiring density. Since this method was suitable for various acquisition densities, the proposed TCO method was based on MVDS, and in view of the good quality of reconstructed images, we compared the effect of polynomial fitting and moving least squares fitting with higher degree on the experimental results, which proved that using the moving least squares method to fit the surface was better. The MVDS method can improve the image quality under the same sampling amount, and this method was combined with MVDS to further optimize the quality of the reconstructed image on the basis of MVDS.

The SENSE method has a rigorous mathematical equation. The sensitivity matrix is ​​directly affects to the quality of the reconstructed image. The more accurate the sensitivity matrix is, the higher the image quality is. In the method proposed in this paper, the existing data was used to continuously optimize the sensitivity matrix to reconstruct images, and results were shown in Fig. 3 and Table 1.

## Conclusion

In summary, we proposed a novel method to achieve a good triple cycle optimization between *k*-space data, the sensitivity matrix and the reconstructed image to improve reconstruction quality in pMRI. This method was suitable for various acquisition densities and had been compared under different acquisition densities. Compared with traditional method, the noise and artifacts were effectively reduced. Combining this method with MVDS greatly improved the image quality, especially when the ACS line was low, the noise and artifacts were significantly reduced. The SENSE algorithm of this method had not changed, but had been optimized on the basis of the original. However, there were many shortcomings. The proposed TCO method was only suitable for SENSE at present, and further improvement is needed to realize a more general algorithm.

## Supplementary Information

Supplementary Information.

## Data Availability

In-vivo data collection was funded from an open dataset at https://people.eecs.berkeley.edu/~mlustig/Software.html (University of California, Berkeley, USA). All Data are available from the corresponding author upon reasonable request.
