# Author Correction: Parallel MR image reconstruction based on triple cycle optimization

**DOI:** 10.1038/s41598-022-17651-9

**Published:** 2022-08-02

**Authors:** Jinhua Sheng, Jie Yin, Luyun Wang, Xiaofan Yang, Pu Huang

**Affiliations:** 1 College of Computer Science, Hangzhou Dianzi University, Hangzhou, 310018 Zhejiang China; 2 Key Laboratory of Intelligent Image Analysis for Sensory and Cognitive Health, Ministry of Industry and Information Technology of China, Hangzhou, 310018 Zhejiang China

Correction to: *Scientific Reports* 10.1038/s41598-022-11935-w, published online 11 May 2022

The original version of this Article contained an error in the spelling of the author Xiaofan Yang which was incorrectly given as Xiaofang Yang.

The original Article has been corrected.

