# Supplementary material for: Parallel MR image reconstruction based on triple cycle optimization
Source: Sci Rep. 2022 May 11;12:7783. doi: 10.1038/s41598-022-11935-w (PMC9095676; doi:10.1038/s41598-022-11935-w)
Supplement: Supplementary file 1 — Supplementary Information. [file 41598_2022_11935_MOESM1_ESM.pdf]

Feb. 7nd, 2022

# Statement

The study was approved by the institutional review board (IRB) at Hangzhou Dianzi University (IRB-2020002) and methods were carried out in accordance with relevant guidelines and regulations. The source data were obtained from an open dataset at https://people.eecs.berkeley.edu/~mlustig/Software.html (University of California, Berkeley, USA). I guarantee that data are permitted to publish in an online open access publication.

1. The **GE_human_brain.mat** file corresponds to the experiment contents (see Fig.1 and attachment 1):

The datasets were acquired on a GE 3T scanner (GE Healthcare, Waukesha, WI) with an 8-channel head coil). The dataset was an axial brain image acquired using a 2D spin echo sequence (TE/TR=11/300ms, matrix size =256×256, FOV = 220 mm×220mm).

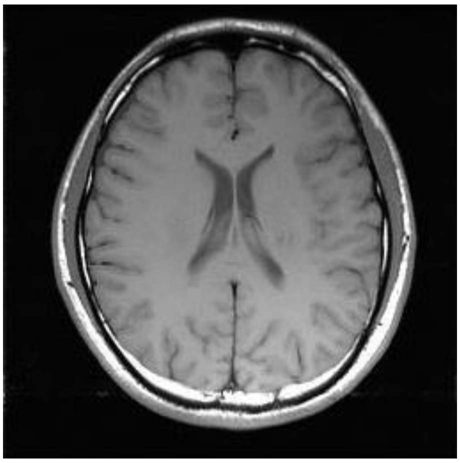

Figure 1. Fully sampled standard image

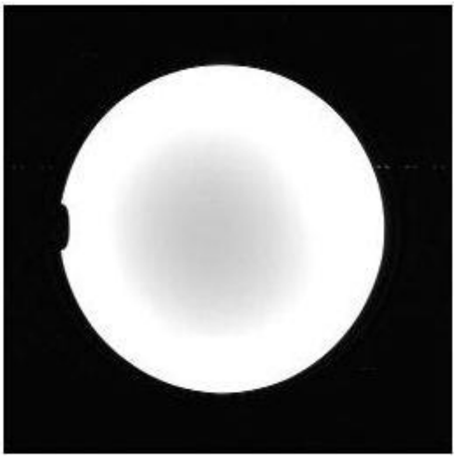

Figure 2. Fully sampled standard image

2. The **circle_phantom_8c.mat** file corresponds to the experiment contents (Fig.2 attachment 1):

The datasets were acquired on a GE 3T scanner (GE Healthcare, Waukesha, WI) with an 8-channel head coil). In this dataset, a uniform water phantom was scanned using a gradient echo sequence (TE/TR = 10/100 ms, 31.25 kHz bandwidth, matrix size = 256 × 256, FOV = 250 mm×250mm).

Sincerely,

**Jinhua Sheng**, Ph.D

Distinguished Professor of Hangzhou Dianzi University, Hangzhou, China.

Director of Key Laboratory of Intelligent Image Analysis for Sensory and Cognitive Health, Ministry of Industry and Information Technology of China, Hangzhou, China.

Senior Member of **IEEE**

**Associate Editor of IEEE Access**

**Editorial Board Member of Scientific Reports**
